# *Lrpap1* deficiency leads to myopia through TGF-β-induced apoptosis in zebrafish

**DOI:** 10.1186/s12964-022-00970-9

**Published:** 2022-10-19

**Authors:** Shanshan Liu, Ting Chen, Binghao Chen, Yijun Liu, Xiaohe Lu, Jiali Li

**Affiliations:** 1 Department of Ophthalmology, Zhujiang Hospital, Southern Medical University, Guangzhou, China; 2 Department of Orthopedics, Guangdong Women and Children Hospital, Guangzhou, China; 3 Department of Foot and Ankle Surgery, Center for Orthopedic Surgery, The Third Affiliated Hospital of Southern Medical University, Guangzhou, China

**Keywords:** LRPAP1, Myopia, Transforming growth factor β, Zebrafish, Apoptosis

## Abstract

**Background:**

Frameshift mutations in *LRPAP1* are responsible for autosomal recessive high myopia in human beings but its underlying mechanism remains elusive. This study aims to investigate the effect of *LRPAP1* defect on ocular refractive development and its involved mechanism.

**Methods:**

A *lrpap1* mutant zebrafish line with homozygous frameshift mutation was generated by CRISPR/Cas9 technology and confirmed by Sanger sequencing. The ocular refractive phenotype was analyzed by calculating the relative refractive error (RRE) with vivo photography and histological analysis at different development stages, together with examining ocular structure change via transmission electron microscopy. Further, RNA sequencing and bioinformatics analysis were performed. The potentially involved signaling pathway as well as the interacted protein were investigated in vivo.

**Results:**

The *lrpap1* homozygous mutant zebrafish line showed myopic phenotype. Specifically, the mutant lines showed larger eye axial length-to-body length in one-month old individuals and a myopic shift with an RRE that changed after two months. Collagen fibers became thinning and disordered in the sclera. Further, RNA sequencing and bioinformatics analysis indicated that apoptosis signaling was activated in mutant line; this was further confirmed by acridine orange and TUNEL staining. Moreover, the expression of TGF-β protein was elevated in the mutant lines. Finally, the treatment of wild-type embryos with a TGF-β agonist aggravated the degree of eyeball apoptosis; conversely, the use of a TGF-β inhibitor mitigated apoptosis in mutant embryos.

**Conclusion:**

The study provides functional evidence of a link between *lrpap1* and myopia, suggesting that *lrpap1* deficiency could lead to myopia through TGF-β-induced apoptosis signaling.

**Video abstract**

**Supplementary Information:**

The online version contains supplementary material available at 10.1186/s12964-022-00970-9.

## Background

Myopia or nearsightedness is characterized by a mismatch between the axial length of the eye and its refractive power, leading to the projection of a defocused image on the retina. This condition is the most common cause of refractive error and has recently emerged as a global health issue [1, 2]; of note, high myopia increases the risk of retinal detachment, glaucoma, cataracts, and myopic macular degeneration [3].

Myopia is influenced by genetic and environmental factors; the contribution of the former has been estimated to be 60%–80% [4]. In fact, molecular genetic studies based on families/populations with high myopia have identified multiple loci and pathogenic genes, including low-density lipoprotein receptor-related protein-associated protein 1 (*LRPAP1*) [5–9]. To date, a total of three homozygous frameshift mutations in *LRPAP1* have been found in fourteen patients with early-onset high myopia from nine unrelated families: c.863_864delTC (p.Ile288Argfs*118) (n = 10) [7, 8], c.605delT (p.Asn202Thrfs*8) (n = 3) [6], and c.199delC, (p.Q67sfs*8) (n = 1), as reported in our previous study [7]. Altogether, these findings indicate a relationship between frameshift mutations in *LRPAP1* and autosomal recessive high myopia. However, the underlying mechanism is still unclear.

LRPAP1 functions as a chaperone of lipoprotein receptor-related proteins [10, 11]. Previous studies have shown that the main functions of LRP1 are to assist the liver in clearing plasma proteins, control the function of adipocytes and the blood–brain barrier, regulate lipid metabolism, and transport the Aβ peptide in Alzheimer’s disease [12] through platelet-derived growth factor and transforming growth factor β (TGF-β) signaling [13, 14]. TGF-β signaling has also been shown to be involved in myopia, particularly in the remodeling of the sclera extracellular matrix [15–17]; the loss of function of LRPAP1 may lead to a decrease in LRP1, which in turn activates TGF-β signaling [9]. However, whether TGF-β was interacted with *LRPAP1* in regulation of myopia development remains unknown.

Zebrafish and humans have 70% sequence similarity, as well as a similar development of the visual systems; therefore, zebrafish is an appropriate model for the study of genetic and environmental eye diseases [18, 19]. Here, we analyzed the role of the *lrpap1* gene, orthologous to the human *LRPAP1* gene by generating a *lrpap1* frameshift mutant. The mutant line with *lrpap1* deficiency resulted in myopic phenotype. Our mechanistic analysis indicated that apoptosis signaling was activated in *lrpap1* mutant zebrafish, in a TGF-β-dependent fashion. Altogether, our results provide new insights into the mechanisms underlying *LRPAP1*-related myopia.

## Materials and methods

### Ethics statement

All experiments involving zebrafish were approved by the Animal Care and Use Committees of Southern Medical University and the South China University of Technology.

### Zebrafish lines and maintenance

The founder of zebrafish line was purchased from the China Zebrafish Resource Center (CZRC Catalog ID CZ921). A stable *lrpap1* homozygous mutant line was obtained via several rounds of crossing and genotyping. Mutalyzer 2.0.34 (https://mutalyzer.nl/) was used to check variant descriptions and predict the affected protein from the variant coding sequence. Protein Homology/analogY Recognition Engine V 2.0 (Phyre2, http://www.sbg.bio.ic.ac.uk/phyre2) was used to predict the protein tertiary structure [20]. All animal procedures were performed according to previously established protocols [21]; embryos were collected and staged as described by Kimmel et al. [22].

### Genotyping

The tail fin tissues of zebrafish were extracted, and each zebrafish was kept in isolation until the genotype was determined. Each tail fin was immersed in 30 μL of 50 mM NaOH and incubated at 96 °C for 40 min. After the tissue was completely dissolved, 3 μL of 1 M Tris–HCl (pH 8.0) was added to the solution (Additional file 1). PCR was performed using ApexHF HS DNA Polymerase FS (Accurate Biology, China) and the primers were: *lrpap1*-Forward: GGATAGCGCTGCAGATGCTC/ *lrpap1*-Reverse: AACTTGCTTCACGTTAACTGCGAGTA. Single bands from agarose gels were sequenced (Shanghai Shenggong Bioengineering Co. Ltd).

### Quantitative real-time PCR (qRT-PCR)

Total RNA was extracted using TRIzol (Accurate Biology, AG21101, Hunan, China), reverse-transcribed using random primers and reverse transcriptase (Yeasen, 11121ES60, Shanghai, China). qRT-PCR was performed on a real-time PCR system (CFX96 Connect, Bio-Rad, USA) using the qPCR SYBR Master Mix (Yeasen, 11203ES03, Shanghai, China). The expression levels were determined with the obtained threshold cycle values using the 2^−△△Ct^ method. β-Actin was used as the housekeeping gene in this study. The primer sequences used were as follows: *lrpap1*-Forward: 5’-GCAACAACCAGGTGGAAT-3’; *lrpap1*-Reverse: 5’-TCAAGTCACTGTGTAGTTCTG-3’; *β-actin*-Forward: 5’-TTCTTGGGTATGGAATCTTGCGGTATC-3’; *β-actin*-Reverse: 5’-CAGTGTTGGCATACAGGTCCTTACG-3’.

### Longitudinal measurements of the eye dimensions via in vivo imaging

Zebrafish were anesthetized with 5 mg/L tricaine (Macklin, E808894, Shanghai, China) and imaged using an Olympus stereoscopic fluorescence microscope from the side and back perspectives. Images were uploaded into Image J (version 1.51) to measure values of the following parameters: axial length (from the front of the cornea to the back of the sclera), body length (from the top of the head to the end of the trunk before the caudal fin), and lens diameter (from the anterior to the posterior surface of the lens). The time points analyzed were 7, 14, and 21 days post-fertilization, as well as one and two months post-fertilization. The relative refractive error (RRE) was calculated [23, 24]: 1 − retinal radius/(lens radius × 2.324), where retinal radius = (axial length—lens radius).

### RRE measurement through the histology method

Heads were fixed in 10% neutral formalin at room temperature (RT) overnight, then placed in EDTA decalcification solution [25]. Samples were dehydrated and embedded in paraffin (Additional file 2: Table S1) to prepare sections of 4-μm thickness (Leica, RM2245, Germany). Central eye sections where the optic nerve is located (or close to) was selected for hematoxylin and eosin (HE) staining (Additional file 3: Table S2). The axial length was measured from the front of the cornea to the back of the retinal pigment epithelium, and the lens diameter was measured from the anterior surface to the posterior surface.

### Electron microscopy of the scleral collagen fibers

The eyeballs of three-month-old zebrafish were processed for electron microscopy as previously described [26] and imaged using a transmission electron microscope (TEM, JEM-1400 PLUS, Japan Electron Optics Laboratory). One hundred and twenty collagen fibers were selected from six different locations in each group, and the fiber diameter and cross-sectional area were measured using the ImageJ software.

### Western blot analysis

Both eyes of each fish were lysed in RIPA lysis buffer (Beyotime, P0013B, Shanghai, China) containing protease inhibitors. The protein concentration was determined using the BCA Protein Assay Kit (Thermo, NO.23227, USA), and samples were resolved on SDS-PAGE and transferred to nitrocellulose membranes. The membranes were blocked in 5% nonfat milk with shaking for 1.5 h at RT, and incubated with primary antibody (1:100 dilution) at 4 °C overnight. After washing the membrane with Tris-buffered saline-Tween (Boster, AR0195, Wuhan, China) three times (10 min each), HRP-conjugated secondary antibody (1:5000, NO. BA1054, Boster, Wuhan, China) was added and incubated for two hours at RT. Finally, proteins were visualized by using an enhanced chemiluminescent detection reagent (Tannon, 180–501, Shanghai, China) and an ECL detection system (Tannon 5200, Shanghai, China). The anti- Lrpap1 primary antibody was prepared in-house via the immunization of rabbits with the peptide SKEMNEKNASDKSNN + Cys. The anti-TGF-beta 1 primary antibody was purchased from SAB (NO.48569, Signalway Antibody, USA).

### RNA-seq and bioinformatics analysis

Twelve pairs of samples (eyeballs from three-month-old zebrafish, including six wild-type and six mutant) were collected. After total RNA was extracted, mRNA with a PolyA tail was enriched with oligo(dT). Then, 6G clean bases were generated by sequencing (Illumina HiSeq2500). RNA-seq reads were aligned to the zebrafish genome (Ensembl, GRCz11) using HISAT2 [27], and TPM was used to further normalize the gene expression counts [28]. Differentially expressed genes were determined using DESeq2, based on the negative binomial generalized linear model using the following cut-off values: |log2 (fold change)|> 1 and adjusted *p *value < 0.05.

### Treatment with the TGF-β agonist or inhibitor

At 20 hpf, wild-type embryos were treated with hydrochloride (10 μM TGF-β agonist, HY-100347A, MedChemExpress, USA), whereas *lrpap1* mutant embryos were treated with oxymatrine (10 μM TGF-β inhibitor, HY-NO158, MedChemExpress, USA). Fresh agonist or inhibitor solution was added once per day.

### Detection of cell apoptosis

Embryos were immersed in 2 μg/mL acridine orange (AO) in E3 medium for 40 min and then rinsed four times with E3 medium. After incubation with 140 mg/L tricaine for three minutes to obtain an effective anesthesia, embryos were observed and imaged using Olympus (SZ61, Olympus, Japan) and Zeiss (SteREO Discovery, V20, Carl Zeiss AG, Germany) microscopes.

For Tdt-mediated dUTP nick-end labeling (TUNEL) immunostaining, the One Step TUNEL Apoptosis Assay Kit (Beyotime Biotechnology, Shanghai, China) was used following the manufacturer’s instructions. After treatment with the fluorescent labeling solution, the cell nuclei were stained with DAPI (Solarbio, Beijing, China) for 10 min. All samples were imaged using an inverted fluorescence microscope (model no. TI2-E, Nikon, Japan).

### Statistical analysis

All data were processed, calculated, and graphed using GraphPad Prism 8. Statistical significance was determined using the Student’s t-test, Linear regression analysis or the one-way ANOVA with Bonferroni correction (more than two groups). The significance levels are defined as follows: ns, *p* > 0.05; *p ≤ 0.05; **p ≤ 0.01; ***p ≤ 0.001; ****p ≤ 0.0001.

## Results

### Characterization of the zebrafish *lrpap1* homozygous mutant line

**Fig. 1:**
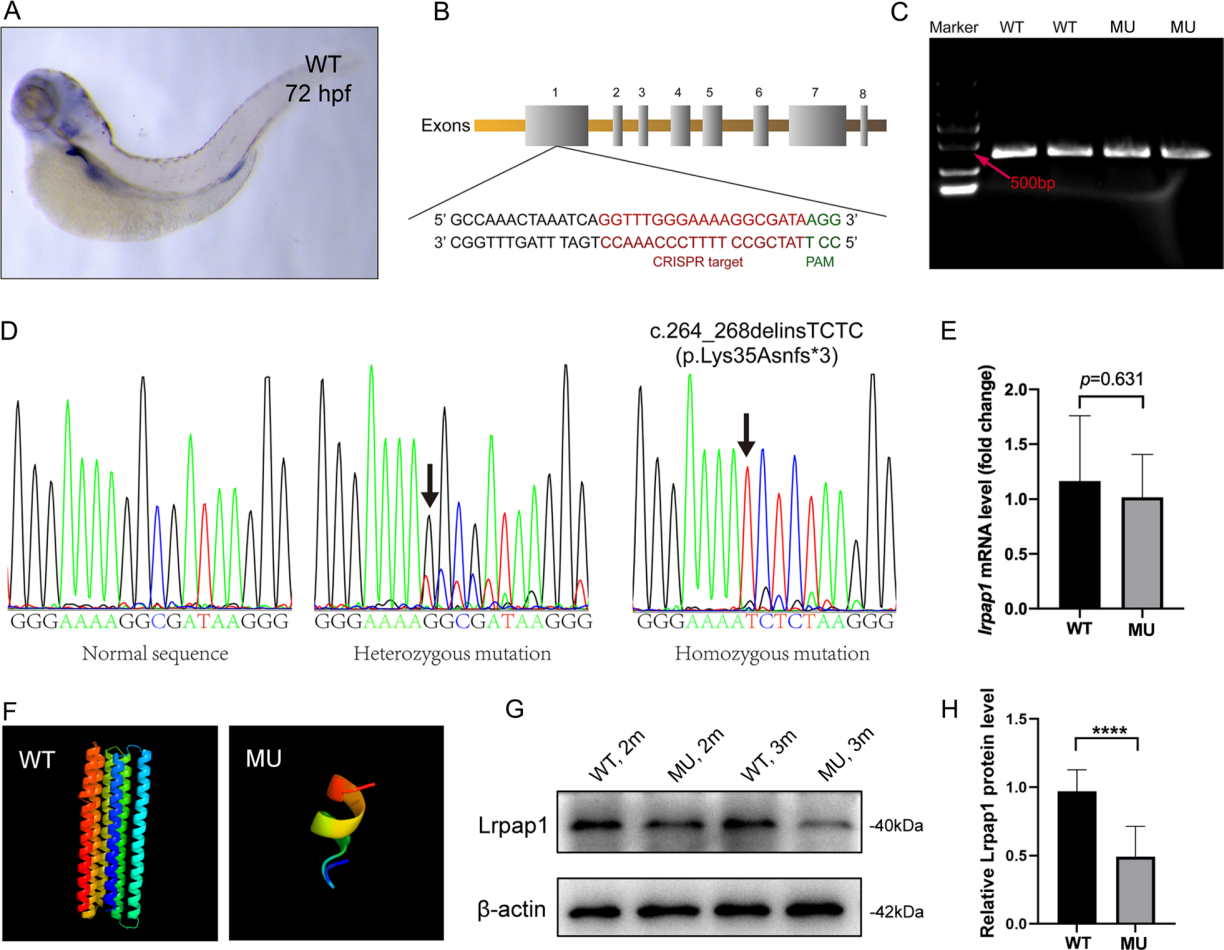
Generation of *lrpap1* knockout zebrafish. **A** Representative image of a zebrafish embryo (72 hpf) subjected to whole-mount in situ hybridization targeting *lrpap1*. **B** The gRNA target and its corresponding position in the *lrpap1* gene sequence are shown. **C** Agarose gel electrophoresis of the *lrpap1* DNA amplified fragments. **D** DNA sequencing results of wild-type, heterozygous, and homozygous zebrafish; the 5 bp deletion/4 bp insertion mutation (c.262_266delinsTCTC) is highlighted by the black arrows. The mutation was predicted to cause a frameshift with premature translation termination, resulting in an abnormal protein (p.Lys35Asnfs*3). **E** *lrpap1* mRNA expression was slightly decreased in the *lrpap* mutant lines, although this was not statistically significant (*p* = 0.631). **F** The protein structure of wild-type and mutant Lrpap1, as predicted by Phyre2. Image colored using the rainbow colors, from the N to C terminus. **G** Western blot analysis of the levels of Lrpap1 in the eyes of *lrpap1* mutant and wild-type zebrafish two months and three months post-fertilization. **H** Relative quantitative result of Lrpap1 protein revealed that the Lrpap1 protein levels were markedly lower in mutant zebrafish than in the wild-type. n = 10 for wild-type zebrafish and n = 10 for mutants*.* Statistical significance was determined using the Student’s t-test. ****p < 0.0001. β-Actin was used as the internal control. WT, wild-type; MU, *lrpap1* homozygous mutant. hpf, hours post-fertilization; 2 m, two months; 3 m, three months

In situ hybridization showed that *lrpap1* is widely expressed in wild-type embryos, 72 hpf, mainly in the kidneys and head, including in the eyeball (Fig. 1A). *lrpap1* frameshift mutations were introduced by CRISPR-Cas9 technology. The CRISPR target and PAM site were in exon1 (Fig. 1B); a 5-base deletion and a 4-base insertion mutation were confirmed by Sanger sequencing (Fig. 1C, D), described as c.264_268delinsTCTC and predicted to result in an abnormal protein (p.Lys35Asnfs*3). Of note, results of qRT-PCR analysis (Fig. 1E) reveal that *lrpap1* mRNA expression was slightly decreased in the *lrpap1 *mutant lines, although this was not statistically significant (*p* = 0.631). Phyre2 model with the highest confidence is shown in Fig. 1F. For wild-type zebrafish, 276 residues (83% of the sequence) were modeled with 97.4% confidence and for the mutant, 12 residues (33% of the sequence) were modeled with 93.5% confidence. Importantly, the predicted tertiary structure of the Lrpap1 protein changed greatly in the context of the *lrpap1* mutation. Western blot analysis revealed that the Lrpap1 protein levels were markedly lower in *lrpap1* mutant zebrafish than in the wild-type, both two- and three-months post-fertilization (Fig. 1G), with the relative quantitation result shown in Fig. 1H (*p* < 0.0001).

### *Lrpap1* deficiency leads to progressive myopia

**Fig. 2:**
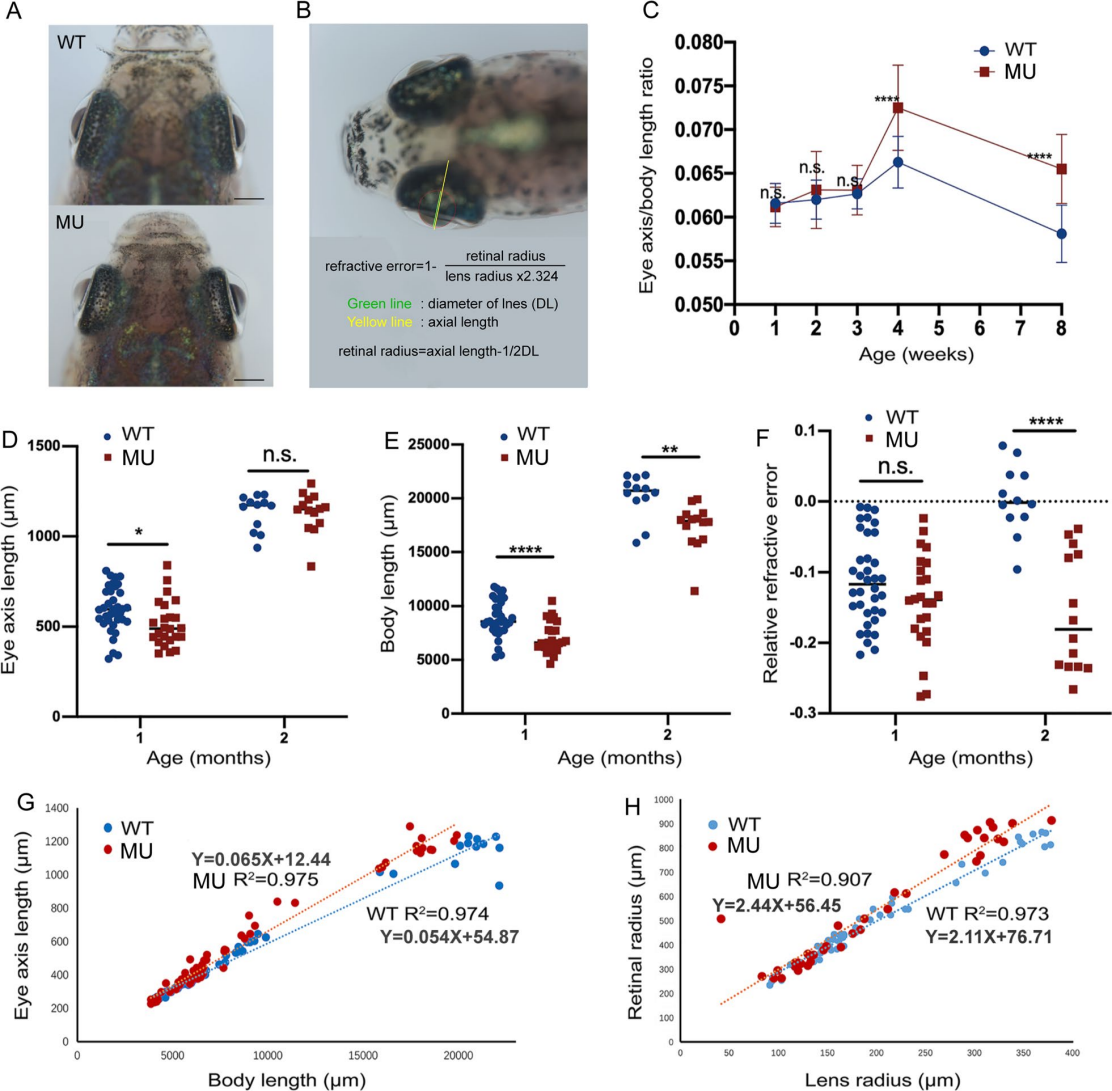
Longitudinal measurements of the eye dimensions using in vivo imaging. **A** Eye globes of wild-type and *lrpap1* mutant zebrafish at three months. The scale bars refer to 500 μm. **B** The different ocular parameters measured and the method of calculation of the relative refractive error (RRE) used. **C** The eye axis to body length ratio was determined at different time points. The eye axis length (**D**) and body length (**E**) of one- and two-month-old wild-type and mutant zebrafish were also determined individually. **F** RRE measurements in one- and two-month-old mutant and wild-type zebrafish. **G** Correlation analysis of the eye axis length and body length measurements. **H** Correlation analysis of the retinal radius and lens radius measurements. WT, wild-type. MU, *lrpap1* homozygous mutant. Statistical significance was determined using the Student’s t-test: n.s. = no significance; *p < 0.05; **p < 0.01; ***p < 0.001; ****p < 0.0001. For wild-type zebrafish, n = 16, 13, 12, 36, and 12 eyes one week, two weeks, three weeks, one month, and two months post-fertilization, respectively. For the *lrpap1* mutant line, n = 12, 10, 13, 24, and 14 eyes one week, two weeks, three weeks, one month, and two months post-fertilization, respectively

Adult zebrafish homozygous mutant line and wild type were shown under in vivo photography, and the heterozygous phenotype has not been investigated in this study (Fig. 2A). To be more specific, different ocular parameters were measured and calculated during the ocular development (Fig. 2B). The eye axis/body length ratio of mutant lines were significantly higher than that of their wild-type counterparts at both one and two months post-fertilization (Fig. 2C), indicating a mismatched development of the eye axis and trunk in mutant lines. Moreover, the eye axis length of *lrpap1* mutant zebrafish was significantly shorter than that of wild-type zebrafish at one month post-fertilization (Fig. 2D), and the body length was significantly shorter in mutant than in wild-type zebrafish at both one and two months post-fertilization (Fig. 2E). In addition, RRE significantly differed between mutant and wild-type zebrafish at two months post-fertilization (− 0.159 ± 0.083 and 0.003 ± 0.049, respectively; Fig. 2F), demonstrating myopic shift was occurred in mutant line. Importantly, consistent with previous study [24], we found a strong linear correlation between the axis of the eye and body length (R^2^ = 0.974, *p* < 0.0001; R^2^ = 0.975, *p* < 0.0001, respectively, Fig. 2G), as well as between the retinal diameter and lens diameter (R^2^ = 0.973, *p* < 0.0001; R^2^ = 0.907, *p* < 0.0001, respectively, Fig. 1H).

**Fig. 3:**
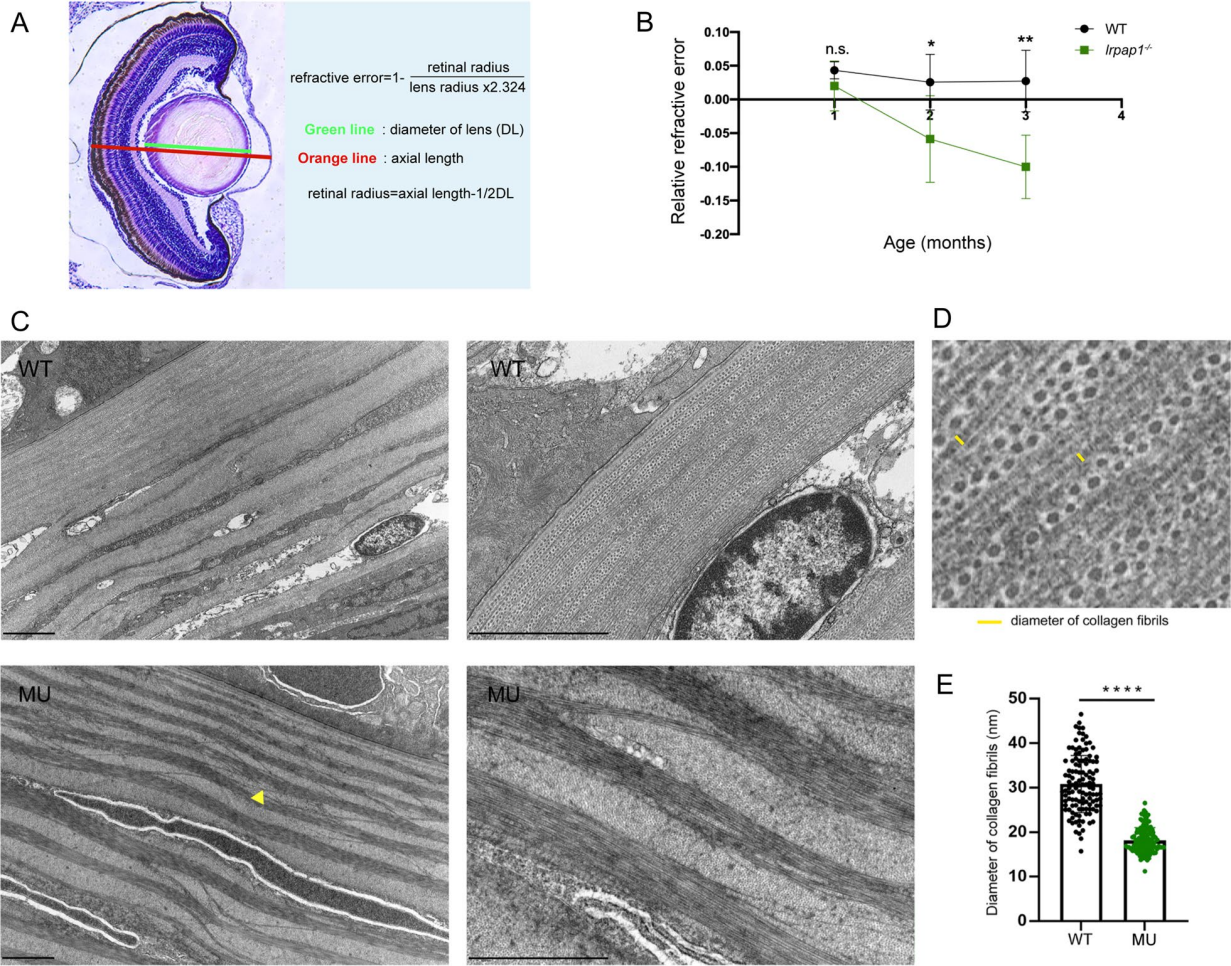
Histological analysis via hematoxylin–eosin (HE) staining and ultrastructural analysis using transmission electron microscopy (TEM). **A** The method used to calculate the relative refractive error (RRE). Refractive error = 1-retinal radius/ (lens radius × 2.324). **B** RRE quantitative data. N = 8 and 14 eyes for 1-month-old wild-type and mutant zebrafish, respectively; n = 18 and 17 eyes for 2-month-old wild-type and mutant zebrafish, respectively; n = 10 and 21 eyes for 3-month-old wild-type and mutant zebrafish, respectively. **C** Electron micrographs of 3-month old zebrafish from the two groups. The yellow triangle points to disordered collagen fibers. The scale bars refer to 2 μm. **D** Schematic diagram of collagen fiber diameter measurement. **E** Quantification of the diameter of the collagen fibers; 120 fibers per group were analyzed. WT, wild-type. MU, *lrpap1* homozygous mutant. Statistical significance was determined using the Student’s t-test: n.s. = no significance; *p < 0.05; **p < 0.01; ***p < 0.001; ****p < 0.0001

We next analyzed the anatomical structure of the zebrafish eyeball using HE staining and TEM. The RRE was calculated based on the anatomical structure (Fig. 3A). The significant RRE difference between mutant and wild-type line were detected from two months post-fertilization, as gradually proceeding toward myopic direction at two and three-months post-fertilization (Fig. 3B), suggesting a progressive myopia development. In line with these results, TEM showed that collagen fibers of sclera were thinner and more disordered in mutant than in wild-type lines (Fig. 3C–E).

### Bioinformatics analysis based on RNA sequencing reveals apoptosis and necroptosis in *lrpap1* mutant line

**Fig. 4:**
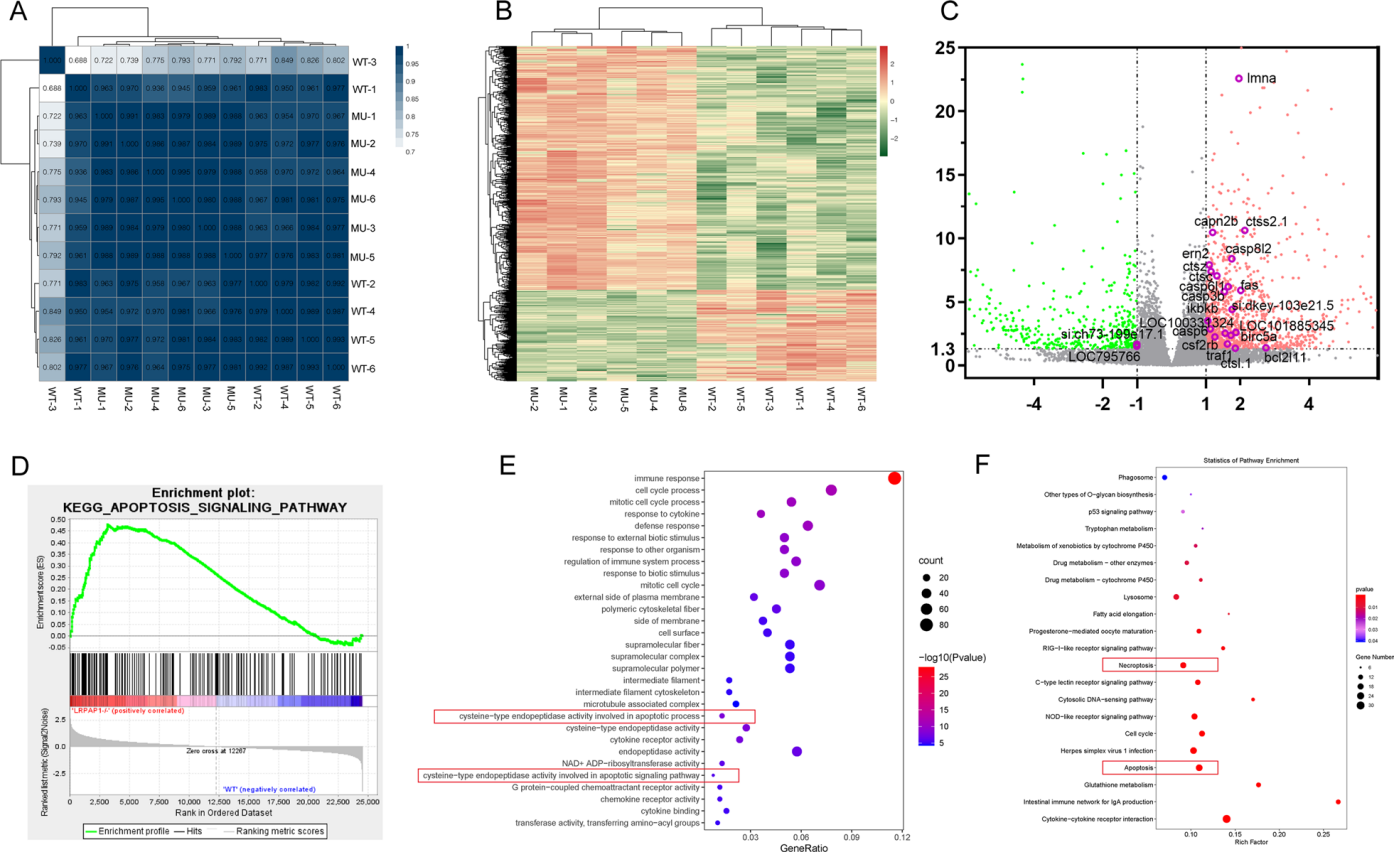
Results of RNA sequencing and bioinformatics analysis. **A** Correlation matrix. The darker the blue, the higher the correlation coefficient. **B** Heat map showing differentially expressed genes in the eyes of *lrpap1* mutant *versus* wild-type zebrafish. The log10 (TPM expression levels + 1) value is color-coded. **C** Volcano plot analysis of differentially expressed genes in *lrpap1*-deficient *versus* wild-type animals at three months post-fertilization. The abscissa represents the change in gene expression multiple in different samples, and the ordinate represents the statistical significance of the change of gene expression. The orange dots represent 91 genes significantly up-regulated in the mutant group, and the green dots represent 247 down-regulated genes. The purple circles show 22 genes associated with apoptosis factors. **D** GSEA analysis in the context of apoptosis pathway gene sets (NES = 1.642, *p* value = 0.010). **E**, **F** GO and KEGG analysis of the differentially expressed (|log2(Fold Change)|> 1 and Q value < 0.05). Apoptotic-related pathways are highlighted with red boxes. WT, wild-type. MU, *lrpap1* homozygous mutant

To identify differentially expressed genes in the *lrpap1* mutants, RNA sequencing was performed on three month-old zebrafish. Based on correlation analysis of 12 samples, expectedly, samples from the same genotype showed a high correlation (Fig. 4A) and this validates our dataset overall. In the heat map (Fig. 4B), each matrix entry represents a gene expression value. Of note, the volcano map (Fig. 4C) shows detection of 1661 differentially expressed genes between *lrpap1* mutant and wild-type zebrafish samples, using the criteria *p*-value < 0.05 and |Log (fold change)|> 1, of which 1209 genes were upregulated and 452 were downregulated. Among them, 22 differentially expressed genes were associated with apoptosis factors, including 20 up-regulated genes and 2 down-regulated genes. We conducted gene set enrichment analysis (GSEA) to examine if the genes in apoptotic signaling pathway were upregulated in the *lrpap1* gene mutant. The results revealed that all the 165 genes in the differential expressed gene set related to apoptosis were enriched at the top of the list; the gene set was upregulated, with an NES score of 1.642 and a *p *value of 0.010 (Fig. 4D, Additional file 4: Table S3). Gene ontology (GO) (Fig. 4E) and Kyoto Encyclopedia of Genes and Genomes (KEGG) analysis (Fig. 4F) were performed. In GO, molecular functions involved in the apoptotic signaling pathway and apoptotic process included endopeptidase activity, and cysteine-type endopeptidase activity (Additional file 5: Table S4). In line with these results, KEGG analysis revealed that many differentially expressed genes were closely related to apoptosis and necroptosis (Additional file 6: Table S5). Detailed results of differential gene expression analysis and the genes of interest are shown in Additional file 5: Tables S4 and Additional file 6: S5, respectively.

### Deleting *lrpap1* triggers apoptosis in the eyes of zebrafish

**Fig. 5:**
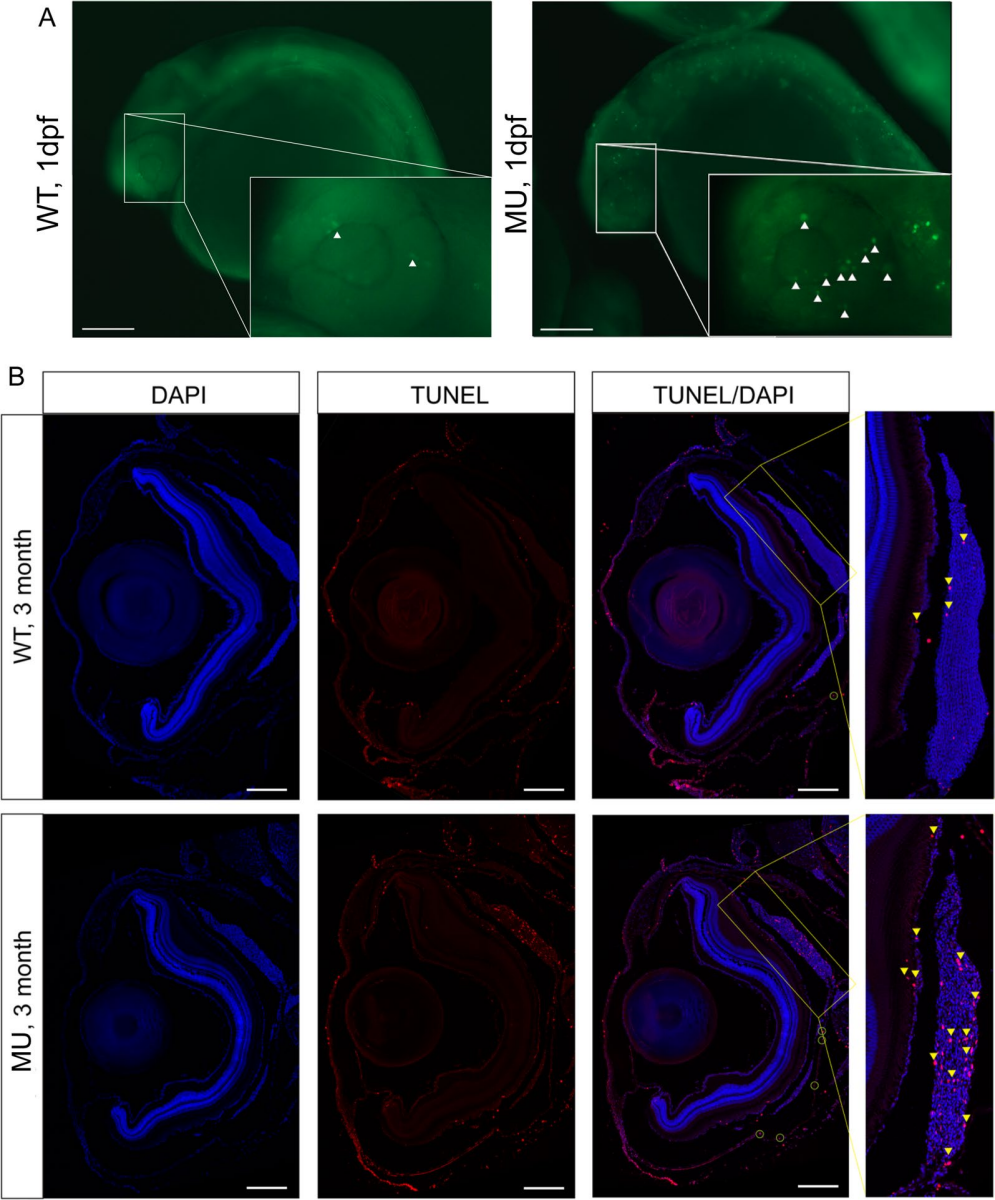
Knockout of *lrpap1* in zebrafish leads to increased apoptosis in the eye. (**A**) AO was used to quantify apoptosis in the eyes of 24 hpf embryos: green fluorescence, as highlighted using white triangles. (**B**) TUNEL staining was also used to investigate ocular apoptosis in adult zebrafish. Apoptosis is highlighted by yellow triangles, particularly in the choroidal tissue (magnified image). The green circles highlight apoptotic cells in the sclera. The scale bars refer to 200 μm. WT, wild-type. MU, *lrpap1* homozygous mutant. dpf, days post-fertilization

AO in vivo staining of embryos 24 hpf revealed many dense yellowish-green fluorescent bodies in the eyes of *lrpap1* mutant zebrafish (Fig. 5A), indicating a higher degree of apoptosis than in the eyes of control animals. The same phenomenon was also observed in adult fish, at three months post-fertilization, as revealed by TUNEL staining (Fig. 5B, red dots). Of note, apoptosis occurred mainly in choroid tissues; a small amount was also detected in the sclera and iris tissues.

### *lrpap1* deficiency is associated with the upregulation of TGF-β

**Fig. 6:**
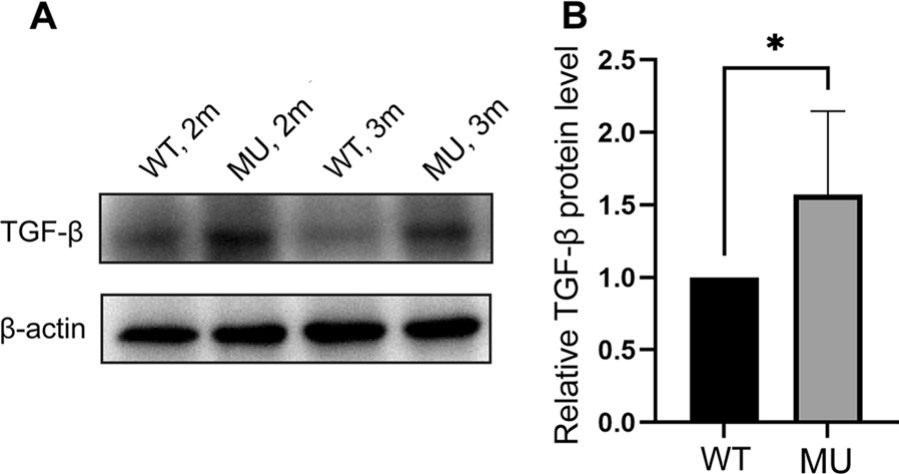
Lrpap1 deficiency is associated with the up-regulation of TGF-β. (**A**) Western blot analysis of TGF-β in the eyes of *lrpap1* mutants and wild-type zebrafish two months and three months post-fertilization. (**B**) Relative quantitative result of TGF-β protein. n = 8 for wild-type zebrafish and n = 8 for mutants*.* Statistical significance was determined using the Student’s t-test. *p < 0.05. β-Actin was used as the internal control. WT, wild-type; MU, *lrpap1* homozygous mutant; 2 m, two months; 3 m, three months

Immunoblot analysis showed that TGF-β was upregulated both at two and three months post-fertilization in mutant compared with wild-type zebrafish (Fig. 6A). Specifically, the relative quantitation result of TGF-β protein was shown in Fig. 6B (*p* < 0.05).

### TGF-β promotes apoptosis in the eye during development

**Fig. 7:**
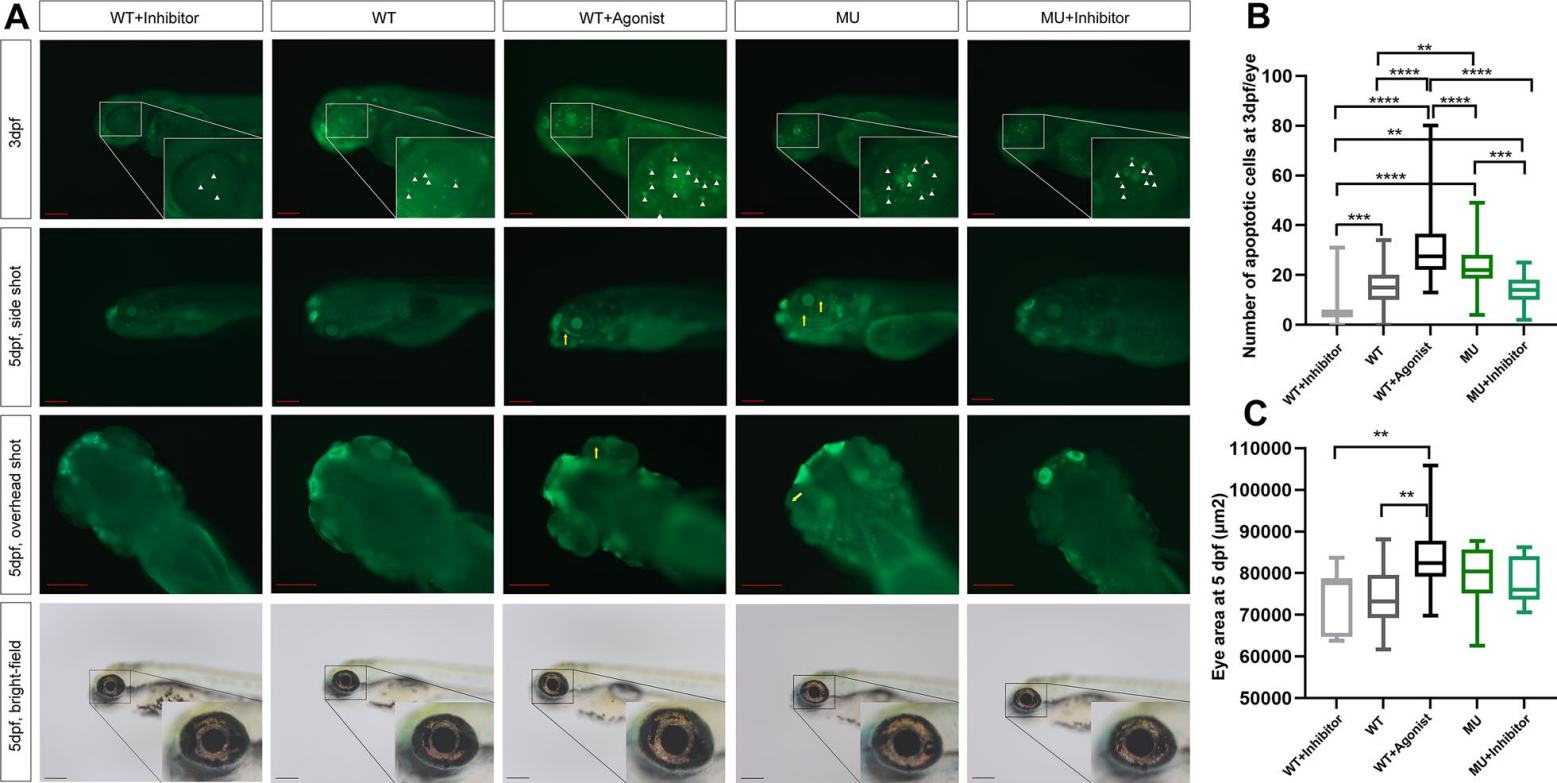
TGF-β promotes apoptosis in the eyes of zebrafish embryos during development. **A** As shown by the white triangles and yellow arrows, the bright green fluorescence from AO staining indicates the apoptosis in eyeballs in each group at 3 dpf and 5 dpf, respectively. The bright-field picture shows the side view of each group of embryos in which the eyeball is magnified in equal proportion. The scale bars refer to 200 μm. **B** Number of apoptotic cells at 3 dpf per eye from AO staining (n = 26, 54, 54, 45, and 39 from left to right). **C** Lateral area of the eyeball in each group (n = 11, 20, 21, 20, and 16 from left to right). *p < 0.05; **p < 0.01; ***p < 0.001; ****p < 0.0001. β-Actin was used as the internal control. WT, wild-type. MU, *lrpap1* homozygous mutant. dpf, days post-fertilization. 2 m, two months. 3 m, three months

Finally, we treated wild-type and knockout zebrafish with TGF-β agonist and TGF-β antagonist, respectively, and analyzed apoptotic cells in the eyes at 3 and 5 dpf. A statistically significant increase in the number of apoptotic cells was observed in wild-type zebrafish treated with the TGF-β agonists (Fig. 7A; B), whereas there was a significant decrease in the number of apoptotic cells in wild-type and mutant zebrafish treated with the TGF-β inhibitor. We found almost no apoptotic cells in the eyes of normal wild-type embryos or embryos treated with antagonist 5 dpf; however, a few apoptotic cells existed in their counterparts treated with the agonist. After the appearance of the *lrpap1* homozygous mutant phenotype, apoptosis persisted in the eyes of zebrafish at 3 and 5 dpf, and even the use of the TGF-β antagonist did not completely eliminate the occurrence of apoptosis. Additionally, at 5 dpf (Fig. 4C), the eyeball area of the agonist-treated group was larger than that of all other groups.

## Discussion

To investigate the role of LRPAP1 in myopia development, a zebrafish model with a homozygous frameshift mutation of *lrpap1* (c.264_268delinsTCTC, p.Lys35Asnfs*3) was generated. A myopic phenotype was characterized in *lrpap1* homozygous mutant line. Furthermore, RNA sequencing analysis and functional studies established a relationship among *lrpap1*, TGF-β, and apoptosis in the context of the development of the eyes.

Indeed, our results showed that the deficiency of the *lrpap1* gene in zebrafish resulted in a myopia phenotype with a higher eye axial length-to-body length ratio, a myopic shift with RRE changes, and thinner scleral collagen fibers. In fact, null mutations of *LRPAP1* play an active role in the myopization process [5–9]. However, thus far, no functional studies have been performed. Interestingly, we found that after *lrpap1* deficiency, zebrafish developed more slowly than their wild-type siblings, as suggested by the fact that the eye axis length of *lrpap1* mutants was shorter than that of wild-type fish. Of note, after adjusting for the trunk length parameters, *lrpap1* mutant line had a larger eye axis length-to-body length ratio, with correlation coefficients close to 1. Therefore, our results, in line with those of previous studies [23, 24], suggest that this ratio is a good index to evaluate the myopia phenotype of zebrafish. In addition, because of the weak refractive power of the cornea in water, the matching degree of the lens size and retinal diameter can be used to evaluate the refractive state [23, 24, 29, 30]. We showed that the diameter of the retina in mutant fish does not match that of the lens; thus, the relatively longer axis of vision causes the image to fall in front of the retina, causing blurred vision. It is worth mentioning that the RRE measured using in vivo imaging and tissue sections was not consistent at one month, which may be owing to a different definition of the retinal radius. The position of the posterior pole of the eye is toward the sclera or choroid, resulting in a larger retinal radius and relative shift in diopter toward myopia. By demonstrating that homozygous mutant of *lrpap1* results in a myopic phenotype in zebrafish, we have created a model with which future studies can explore the mechanism of interaction between genes and environmental factors that causes myopia.

It is intriguing to consider that the loss of *lrpap1* function is associated with the upregulation of TGF-β expression, previously reported as an important molecular change in the process of myopia [16, 31–33]. LRPAP1 has a specific functional domain that binds to the ligand-binding site of LRP1, thereby protecting the LRP1 protein from degradation during folding, maturation, and transportation in cells [34, 35]. Interestingly, a decrease in LRP1 can over-activate the release of TGF-β from extracellular matrix (ECM) sources [36]. In addition, LRP1 regulates the proliferation and migration of human hepatic stellate cells via the modulation of the phosphorylation of ERK1/2 and of the TGF-β extracellular levels [37]. Here, we found that *lrpap1* deficiency led to the upregulation of TGF-β expression. TGF-β plays a role in regulating the ECM and acts in many diseases, including myopia [38–40]. Previous studies have shown that TGF-β is downregulated in the context of scleral tissues with myopia and associated with ECM degradation and fibroblast transformation via the increase in levels of MMP2 or α-SMA [41–43]. However, bFGF and TGF-β were reported to jointly regulate ocular development in a chick model; TGF-β may be an inhibitor of bFGF, leading to axial elongation and diopter change [44]. In our embryo study, treatment of wild-type animals with a TGF-β agonist resulted in faster eye development, which manifested as a larger eyeball area. Zebrafish are a good model for studying the effects of drugs on development; therefore, future studies should explore the effects of drugs or factors on eyeball development [45].

An important question is whether the functions and regulatory mechanism of TGF-β in ocular enlargement identified in this study are related to the activation of apoptosis. TGF-β was found to induce apoptosis in mouse mammary epithelial cells; this could be inhibited by transcriptional co-regulators [46]. Similarly, some studies on TGF-β-induced apoptosis in the context of other cell types have been reported [47]. Here, we discovered that TGF-β increased levels of apoptosis in the eyes and accelerated ocular development in early juvenile zebrafish, suggesting that the nearsightedness phenotype caused by *lrpap1* deficiency may be related to TGF-β-induced apoptosis. Furthermore, we observed sustained apoptosis in *lrpap1* mutant line, unless TGF-β-signaling was inhibited. Of note, myopia in mutant line appeared about one month after birth and became progressively worse, and apoptosis was observed as early as in the embryonic stage, suggesting that continuous apoptosis is the cause rather than just the result of myopia. This being said, we did not explore in depth the specific apoptosis-related molecular changes influenced by TGF-β, and we plan to follow these approaches in a subsequent study.

We hypothesized that the occurrence of apoptosis might interfere with the refractive state, through impact on the choroidal tissue. Recent studies have highlighted the role of the choroid in scleral remodeling and myopia [48]. Some studies have shown that choroidal thickness is negatively correlated with diopter measurements, and the thicker the choroid, the shorter the ocular axis [49, 50]. In this study, TUNEL immunofluorescence showed that apoptosis mainly occurred in the choroid tissue, with lower levels in the sclera and iris. It is possible that apoptosis occurring in the choroid affects the focus of refraction through histomorphological changes or that the activation of the apoptosis pathway indirectly regulates scleral growth remodeling by interfering with the molecular network of growth factors normally secreted by the choroid. All in all, TGF-β-induced apoptosis is a good entry point for future in-depth exploration of retino-choroidal scleral axis signaling network regulating refractive development, thus elucidating the underlying signaling pathways and mechanisms is essential to promote the development of novel therapeutic approaches for myopia based on the modulation of the choroidal function.

## Conclusion

In this study, we found that *lrpap1* gene deficiency leads to the development of a myopic phenotype in zebrafish, which may be related to apoptosis induced by the upregulation of TGF-β.

## Supplementary Information

**Additional file 1**.** Figure S1**. Immunohistochemical (IHC) results of LRPAP1 in 3-month-old zebrafish. (A) Negative control. (B) IHC targeting LRPAP1 for wild-type zebrafish.** Figure S2**. Western blot analysis of LRPAP1 in the eyes of lrpap1 mutants and wild-type zebrafish two months and three months post-fertilization. WT, wild-type; MU, lrpap1 homozygous mutant.** Figure S3**. Western blot analysis of TGF-β in the eyes of lrpap1 mutants and wild-type zebrafish two months and three months post-fertilization. WT, wild-type; MU, lrpap1 homozygous mutant.

**Additional file 2**.** Supplementary Table S1**. Tissue gradient dehydration procedure before paraffin section.

**Additional file 3**.** Supplementary Table S2**. Hematoxylin-Eosin (HE) staining procedure.

**Additional file 4**.** Supplementary Table S3**. Information of 165 apoptosis pathway related genes in GSEA analysis.

**Additional file 5**.** Supplementary Table S4**. Gene information of GO analysis.

**Additional file 6**.** Supplementary Table S5**. Gene information of KEGG analysis.

## Data Availability

All data are included in the article or available on request by contacting the corresponding author: ljli@mail2.sysu.edu.cn. The RNA-seq data have been deposited at the NCBI Gene Expression Omnibus (GEO), and are accessible under the GEO Series accession number GSE186889.
