# Supplementary material for: Lrpap1 deficiency leads to myopia through TGF-β-induced apoptosis in zebrafish
Source: Cell Commun Signal. 2022 Oct 19;20:162. doi: 10.1186/s12964-022-00970-9 (PMC9580148; doi:10.1186/s12964-022-00970-9)
Supplement: Supplementary file 2 — Additional file 1. Figure S1. Immunohistochemical (IHC) results of LRPAP1 in 3-month-old zebrafish. (A) Negative control. (B) IHC targeting LRPAP1 for wild-type zebrafish. Figure S2. Western blot analysis of LRPAP1 in the eyes of lrpap1 mutants and wild-type zebrafish two months and three months post-fertilization. WT, wild-type; MU, lrpap1 homozygous mutant. Figure S3. Western blot analysis of TGF-β in the eyes of lrpap1 mutants and wild-type zebrafish two months and three months post-fertilization. WT, wild-type; MU, lrpap1 homozygous mutant. [file 12964_2022_970_MOESM2_ESM.docx]

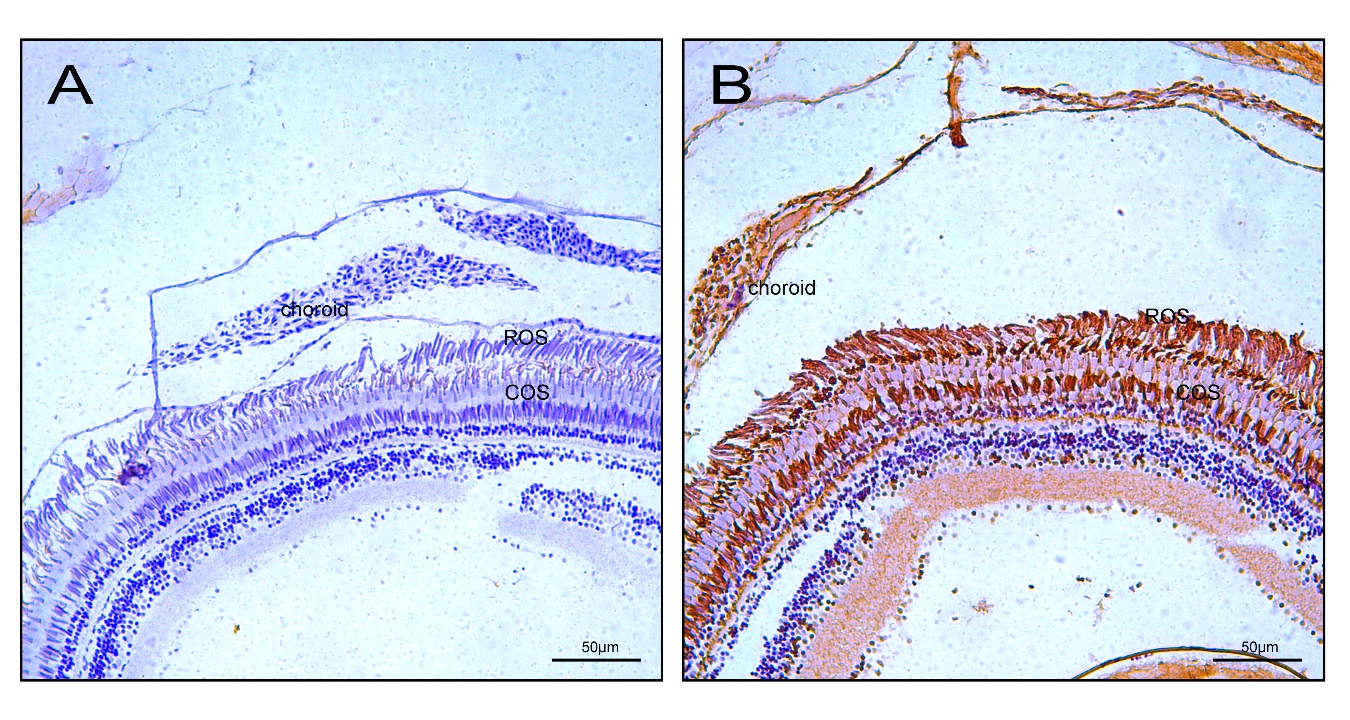
Figure S1. Immunohistochemical (IHC) results of LRPAP1 in 3-month-old zebrafish. (A) Negative control. (B) IHC targeting LRPAP1 for wild-type zebrafish.


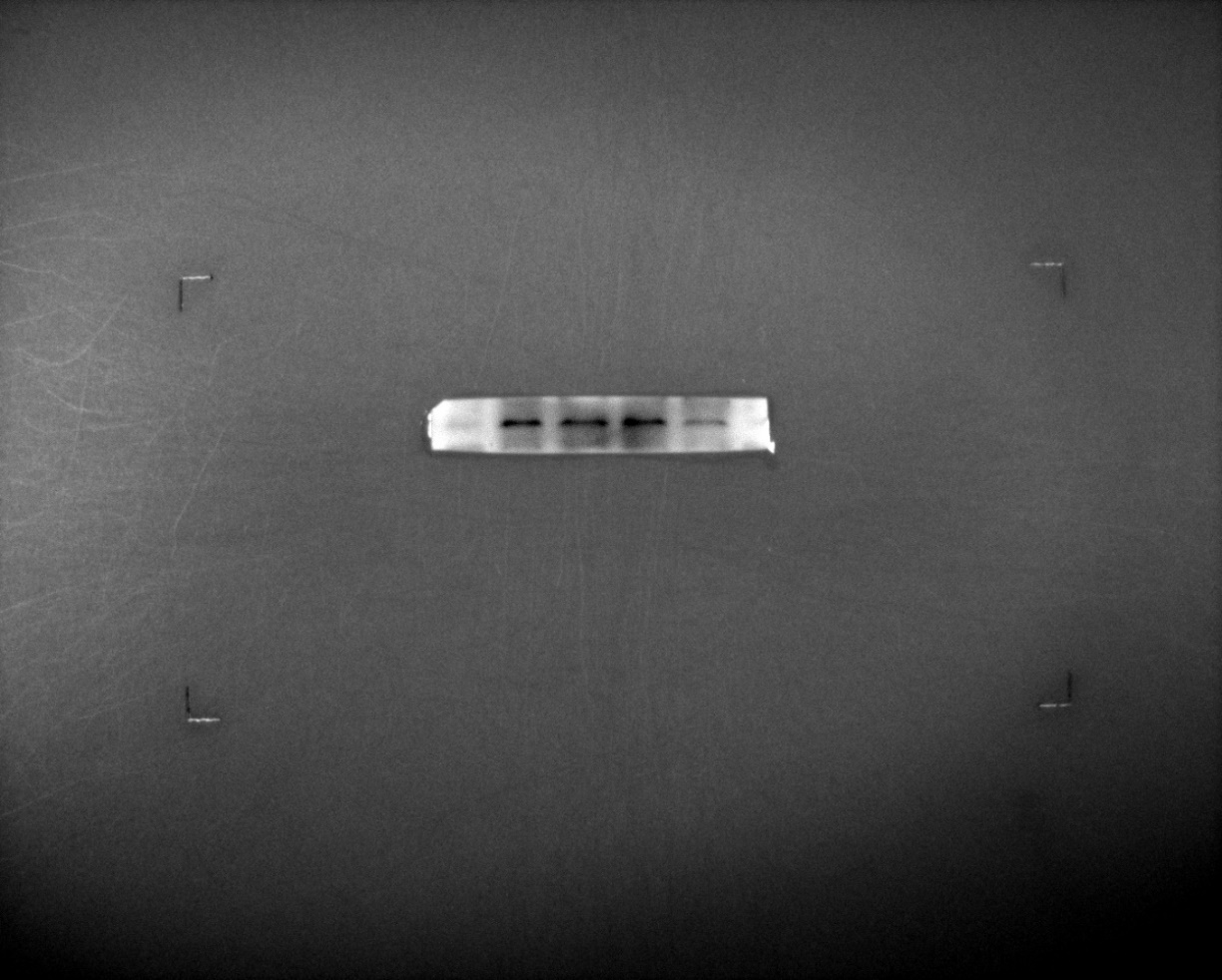

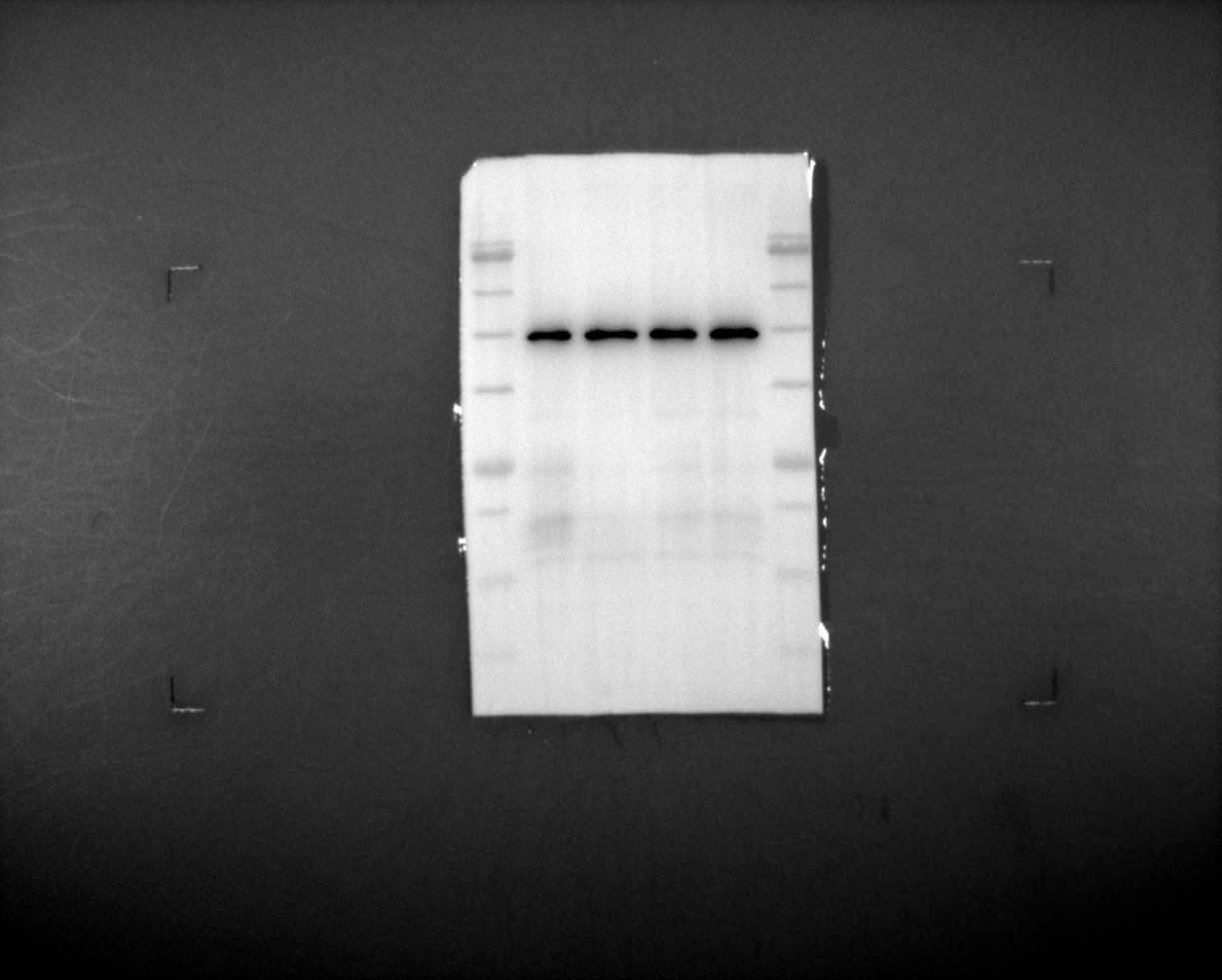


WT，3m

MU，3m

MU，2m

WT，2m

45kDa

35kDa

45kDa

MU，3m

WT，3m

MU，2m

WT，2m

β-actin

Lrpap1

Figure S2. Western blot analysis of LRPAP1 in the eyes of *lrpap1* mutants and wild-type zebrafish two months and three months post-fertilization. WT, wild-type; MU, *lrpap1* homozygous mutant.

β-actin


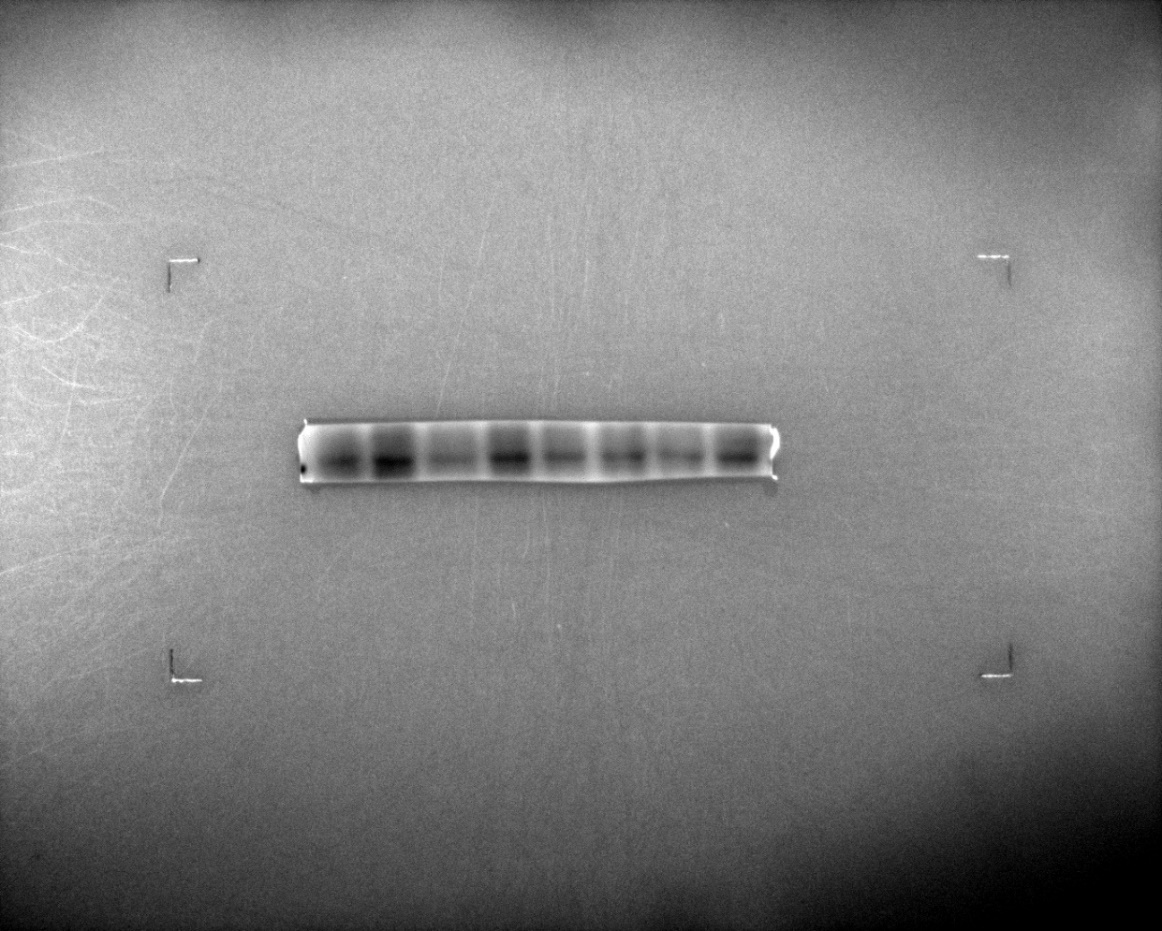

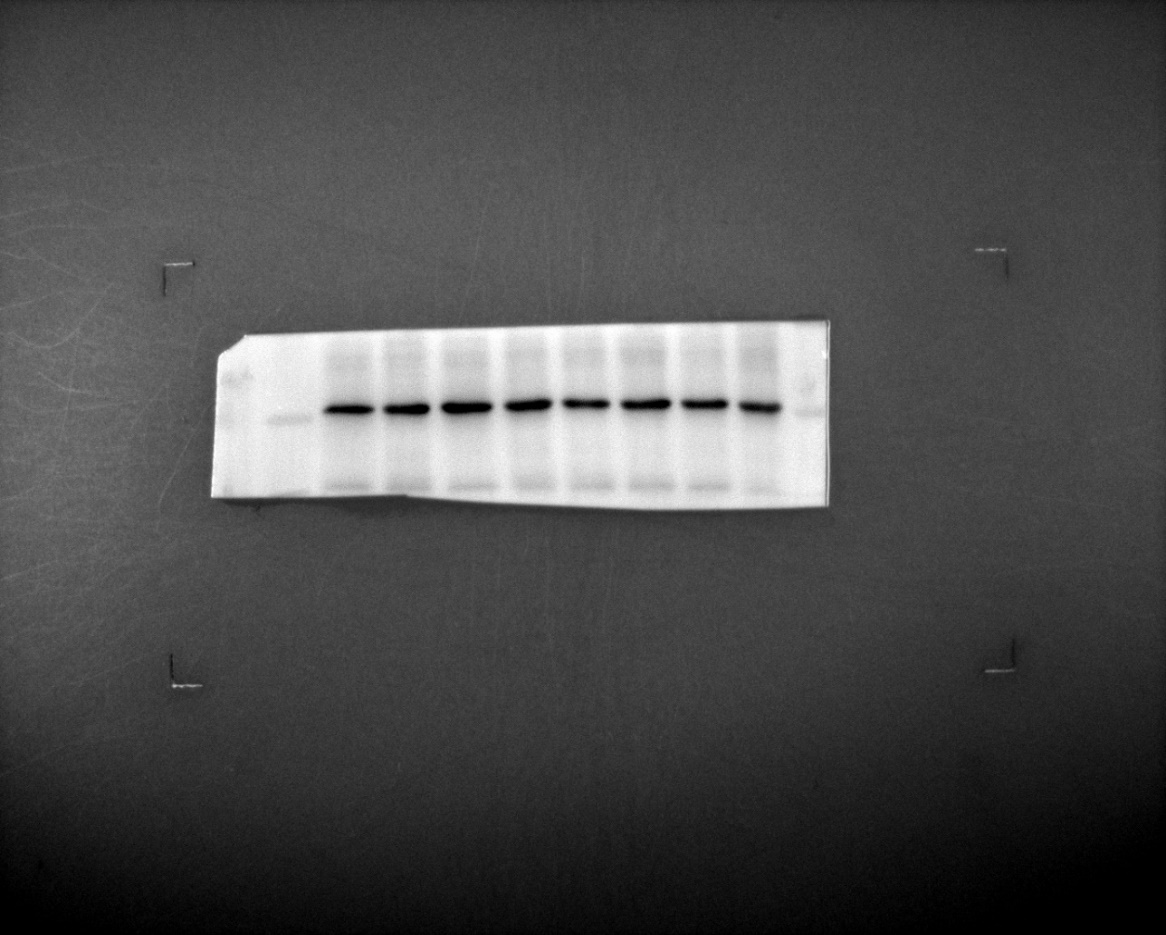


45kDa

MU，3m

WT，3m

MU，2m

WT，2m

MU，3m

WT，3m

MU，2m

WT，2m

MU，3m

WT，3m

MU，2m

WT，2m

MU，3m

WT，3m

MU，2m

WT，2m

TGF-β

45kDa

Figure S3. Western blot analysis of TGF-β in the eyes of *lrpap1* mutants and wild-type zebrafish two months and three months post-fertilization. WT, wild-type; MU, *lrpap1* homozygous mutant.
